# Samarium: from a distorted-fcc phase to melting under dynamic compression using in-situ x-ray diffraction

**DOI:** 10.1038/s41598-022-21332-y

**Published:** 2022-10-06

**Authors:** Sakun Duwal, Chad A. McCoy, Daniel H. Dolan III, Cody A. Melton, Marcus D. Knudson, Seth Root, Richard Hacking, Bernardo Farfan, Christopher Johnson, C. Scott Alexander, Christopher T. Seagle

**Affiliations:** 1 Sandia National Laboratories, Albuquerque, NM 87125 USA; 2 Mission Support and Test Services, Albuquerque Operations, Albuquerque, NM 87125 USA

**Keywords:** Phase transitions and critical phenomena, Structure of solids and liquids, Materials science

## Abstract

Lattice and electronic structure interactions for *f*-electrons are fundamental challenges for lanthanide equation of state development. Difficulties in first-principles calculations, such as density functional theory (DFT), emphasize the need for well-characterized experimental data. Here, we measure in-situ x-ray diffraction of shocked samarium (Sm) and temperature along the Hugoniot for the first time, providing direct evidence for phase transitions. We report direct evidence of a distorted fcc (dfcc) phase at 23 GPa. Shocked samarium melts from the dfcc phase starting at 33 GPa (1333 K), with complete melt at 40 GPa (1468 K). Previous work indicated shock melt at 27 GPa (1200 K), underscoring the significance of x-ray measurements for detecting phase transitions. Interestingly, our observed melting is in sharp contrast with the melting reported by a diamond anvil cell study. These experimental data can tightly constrain first principles calculations and serve as key touchstones for equation of state modeling.

## Introduction

Constraining phase dynamics and melting in lanthanides with in-situ techniques offers a unique opportunity to study the interplay between complex electronic and lattice structures. Equation of state (EOS) measurements combined with phase information are essential to understanding material properties at various conditions, shedding light on the effect of *f*-electrons on material properties. Recent studies on cerium^1^ and samarium monochalcogenides^2^ demonstrate the difficulties in modeling lanthanides with first-principles methods due to their complex electronic and lattice structures. As such, direct structural insights play a vital role in understanding the underlying physics. Traditional shock wave experiments rely on the change in the slope of the shock and particle velocities to infer phase transitions, which can be misleading without direct structural evidence. The recent application of advanced light sources to dynamic x-ray diffraction enable observation of real-time structural changes and melting^3–10^ in a way that until now was out of reach.

Many lanthanides (La to Lu), follow a similar sequence of phase transformations under increasing pressure: hexagonal closed packed (hcp) $\rightarrow$Sm-type $\rightarrow$ double-hcp(dhcp) $\rightarrow$ face-centered cubic (fcc) $\rightarrow$ distorted-fcc (dfcc). In the case of Samarium (Sm), a transition sequence (Pearson notation) from Sm-type *(hR9)*$\rightarrow$ dhcp *(hP4)*$\rightarrow$ fcc *(cF4)*$\rightarrow$ dfcc *(hR24)* $\rightarrow$*hP3* is observed at increasing pressure^11,12,12,13^. Previous shock experiments on Sm interpreted a kink in the shock-particle ($U_s-u_p$) velocity at 27 GPa to be a signature of melting. This is 1000 K lower than the melting curve proposed by Diamond Anvil Cell (DAC) studies, emphasizing the need for in-situ x-ray diffraction and temperature data to identify solid-solid phase transformations and melting^14^. Furthermore, previous shock data^15^ fails to distinguish between any other solid-solid phase transitions, as the measured $U_s-u_p$ curve is insensitive to small volume changes.

This paper presents the first dynamic structural study of Sm up to a maximum pressure of 75 GPa along the principal Hugoniot through melting, showing contrasting, but precise, evidence of a complete melt at 40 GPa. We observe the existence of a solid dfcc phase at 23 GPa, which remains stable up to 33 GPa. Importantly, the direct structural evidence observed in this study further clarifies that the kink observed in the $U_s$ – $u_p$ at 27 GPa in previous study (interpreted as melting)^15^ is in fact a solid-solid transition. The onset of melting actually occurs at 33 GPa and completes by 40 GPa. These results provide important focal points to tightly constrain first principle calculations, allowing valuable insights in understanding material properties in *f*-electron systems.

## Results

### Structural results: X-ray diffraction measurements

**Figure 1:**
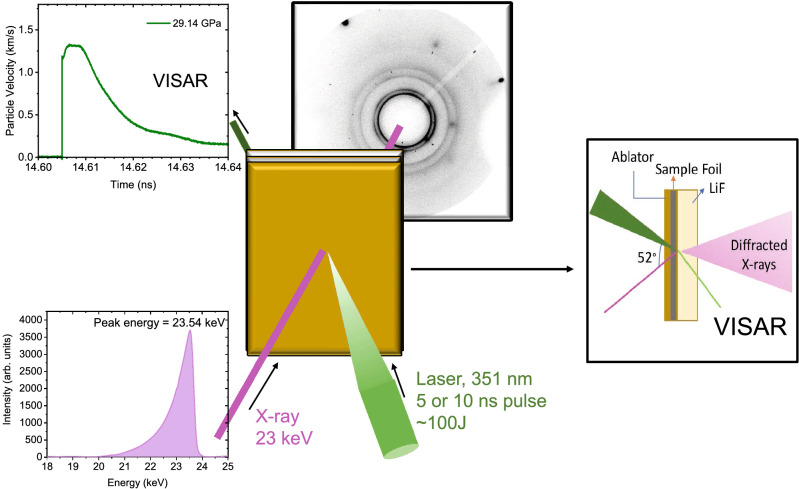
Experimental configuration arranged in a transmission geometry at the DCS laser station showing the pink (non-monochromatic) beam profile of the U17 undulator x-ray source, together with XRD diffraction ring from the Sm sample and processed point-VISAR data collected at the Sm/LiF interface (time zero corresponds drive laser impact). Bright spots correspond to reflection from LiF single crystal. The target assembly consists of kapton ablator backed by Sm foil, which is backed by a LiF window.

**Figure 2:**
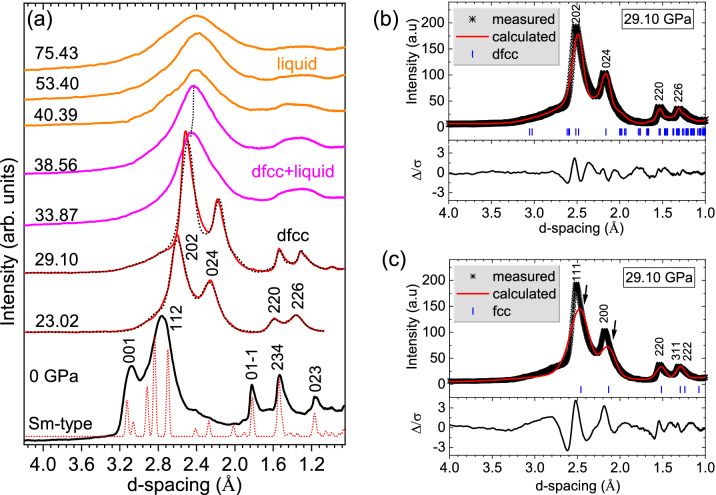
(**a**) Representative x-ray diffraction patterns for different solid, solid+liquid and liquid phases. At 0 GPa, the red dotted line is the simulated x-ray diffraction pattern for Sm-type ambient phase. At 23 and 29 GPa, dotted lines correspond to the Rietveld refined fitting result. (**b**) Rietveld refinement results at 29.1 GPa showing the observed and calculated patterns, reflection tick marks (blue vertical lines) and difference profiles ($\Delta /\sigma$) shown below the refinement curve) using dfcc, *hR24* structure *(R-3m)*. The structural parameters of *a* = 6.18 ± 0.05 Å, *c* = 15.36 ± 0.06 Å, and atomic coordinates of (0, 0, 0.251), and (0.509, -0.509, 0.244) were used for the dfcc phase. (**c**) Rietveld refinement result using the fcc *(Fm-3m)* phase. The structural parameters of *a* = 4.47 ± 0.06 Å, and atomic coordinates of (0, 0, 0) were used. The poor fitting results using the fcc structure clearly suggest that dfcc is indeed the stable phase.

We have performed several experimental runs at the Dynamic Compression Sector (DCS), at the Advanced Photon Source (APS) using the experimental setup shown in Fig. 1. The experimental methods and techniques used for pressure determination are reported in the Methods section. We measured real time x-ray diffraction of laser-shocked samarium samples. We used a 100 J laser to generate shock in the material. Our x-ray diffraction results show clear signatures of a solid-solid phase transition, followed by melting at higher pressure. Figure  2a shows the x-ray diffraction patterns at different pressures. The measured x-ray diffraction at ambient conditions is consistent with the simulated Sm-type phase of Sm (*R-3m*)^16^. Note that the diffraction patterns observed in the current study exhibit characteristic peak broadening compared to the simulated patterns (dotted patterns), due to the width and asymmetry of the pink x-ray (non-monochromatic) beam. Nonetheless, all of the diffraction peaks corresponding to the Sm-type phase are still present.

Upon shock loading Sm to 23 GPa, a different solid phase is observed. The measured diffraction pattern suggests a presence of dfcc phase. Since at lower pressure an fcc phase of Sm is stable, we performed Rietveld refinement using the fcc structure, as well. Interestingly, the refinement results do not favor an fcc phase. Upon using dfcc structure for refinement, the overall fit is significantly improved, hence indicating that dfcc is the favored phase between the two. On the other hand, when fcc structure is used, several mismatches in (i) the width of the intense peak at 2.5 Å, (ii) intensities of the peak at 2.5, 2.2 and 1.5 Å, and (iii) the underestimation in the resultant fits of the overall pattern suggest that fcc structure is not the best candidate for the pattern at 29.1 GPa. This suggests that distorted-fcc is the favored state at these pressures.

**Figure 3:**
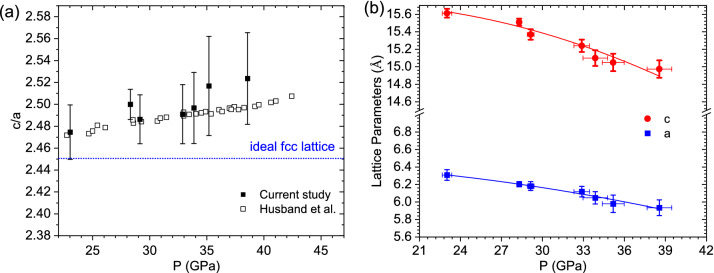
(**a**) c/a ratio for the dfcc phase. The dotted line represents the expected c/a ratios for an ideal fcc phase. Solid symbols are data from our current study, open symbols are data from Husband *et al.*^11^. The large error bar in our data is due to the broader peak features owing to the pink beam. (**b**) Calculated lattice parameters from the diffraction results.

Another clear signature to support the determination of a distorted fcc structure rather than an ideal fcc structure is that the c/a ratio determined from the experimental data is greater than 2.45. Figure 3a plots the *c/a* ratio obtained from this study. The *c/a* ratio continuously increases with increasing pressure, which is consistent with the results from DAC studies^11^. If this structure were to be identical to an ideal fcc material with atoms located at (0, 0, 0.25) and (0.5, 0.5, 0.25), the expected c/a ratio would be $\sqrt{6}$ (2.45). The fact that our c/a ratio deviates from the ideal 2.45 (Fig. 3a), and Rietveld refined atomic coordinates of (0, 0, 0.251), and (0.509, -0.509, 0.244) provides strong evidence that, at 23 GPa and above, Sm is in a distorted fcc phase. We would like to note that a clear signature for the dfcc phase would be the splitting of the [111] fcc peaks in Fig. 2c. However, due to the resolution limited by the pink beam, we are unable to resolve that splitting. Nevertheless, here we rely on the overall improved Rietveld refinement results upon using the dfcc phase, and the increasing *c/a* ratio deviation with increase in pressure, to conclude the favorability of the dfcc phase over the fcc phase.

Figure 3b plots the lattice parameters determined using the x-ray diffraction data. We observe a decrease in the lattice parameters with increasing pressure, which suggests the stiffening of the lattice. The *c*-axis seems to be more sensitive to change in pressure than the *a*-axis, which makes sense as the lattice is stacked along the *c*-direction.

**Figure 4:**
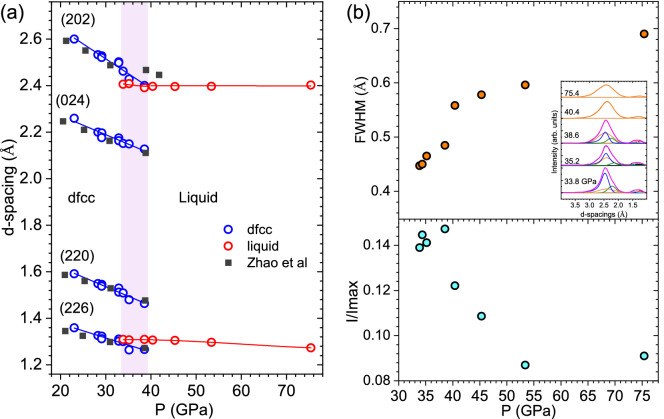
(**a**) d-spacings of four strongest reflections of high pressure phases as a function of pressure. Blue circles correspond to the dfcc solid phase, whereas the red circles correspond to the liquid phase. Solid squares are from Zhao *et al.*^17^ DAC studies. The pink shaded section is where dfcc+liquid phase co-exist with each other. Blue and red solid lines are fits to the data. (**b**) The bottom graph shows the relative intensity ratio of the broad peak at 1.35 Å (I), with respect to the strong broad peak at 2.4 Å ($I_{max}$). The top graph shows the measured FWHM of the intense peak contributing from the liquid phase (orange peaks in the inset) at 2.4 Å. The deconvolution of peaks showing the contribution from solid and liquid phase is presented in the inset.

Figure 4a shows d-spacings as a function of pressure. The deconvolution of peaks suggests that a mixture of dfcc+liquid phase exists between 33 to 40 GPa. Along the Hugoniot, the dfcc phase remains stable up to 32.9 GPa. We observe a decreasing shift in the d-spacings of the lattice planes (Fig. 4b) with increase in pressure due to stiffening of the lattice. The observed d-spacings shows good agreement with Zhao *et al.*^17^ data at ambient temperature. Note that only selected major lines from Zhao *et al.*^17^ data is shown. At 33.8 GPa, we observe an abrupt disappearance of the crystalline features, indicating the presence of a new phase. Rather than sharp peaks, two broad features at 2.4 Å and 1.3 Å are observed, consistent with the diffuse scattering expected from a liquid, suggesting an onset of melting.

We present additional evidence for the co-existence of the solid-liquid phase in the behavior of the Full-Width-Half-Maximum (FWHM) and the intensity ratios (Fig. 4b) of these two broad features contributing from the liquid phase (orange peaks in Fig. 4b inset). At 40.4 GPa, we observe (i) a sudden jump in the FWHM of the peak at 2.4 Å, suggesting that the liquid phase has become dominant; (ii) an abrupt decrease in the relative intensity of the peak at 1.3 Å, suggesting a significantly reduced contribution from the solid (220) and (226) dfcc phases. Finally, we note that the intensity ratio at 53 and 75 GPa are markedly similar.

Upon deconvolution of the features (Fig. 4b inset), the peaks contributing from the solid phase are still present. However, using just the solid phase, the resultant fit was unable to match the measured pattern. Therefore, a liquid profile is also incorporated to the fit. The resulting overall fit shows good agreement with the measured line profile, suggesting that the patterns at 33.9 and 38.6 GPa consist of mixtures of the solid dfcc and the liquid phase. A combination of exponentially modified Gaussian and Lorentzian profiles were used for the least squares fitting of the profiles^5,10^. The peaks contributing from the ambient phases were taken into account. Based on the presence of a much narrower and more symmetrical peak between 33-38 GPa, we conclude that a solid dfcc phase and liquid phase co-exist over this regime. In contrast, above 40 GPa, much broader peaks indicate the presence of a fully melted phase. In this study, we observe clear diffraction patterns with both solid and liquid scattering contributions between 33 and 40 GPa, suggesting the co-existence of solid and liquid.

### Pyrometry results: temperature measurements

**Figure 5:**
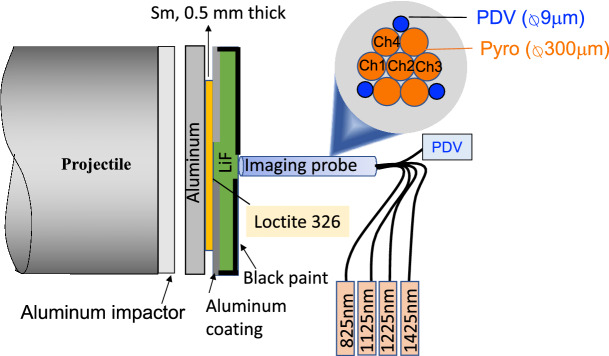
Plate-impact experiment schematic.

**Figure 6:**
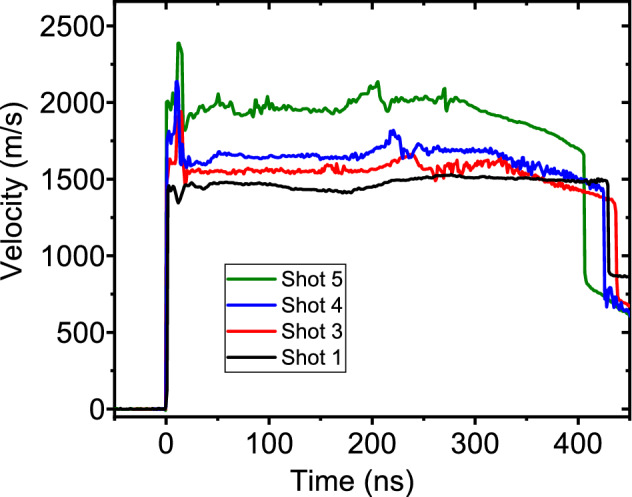
Measured Sm-LiF interface velocities in plate impact experiments. Time zero corresponds to shock breakout time.

Figure 5 illustrates the two-stage, light-gas gun experiments used to obtain Hugoniot data in *P*-*T* space (Table 2); Figure 6 shows how velocity (and therefore pressure) evolved in these experiments. At shock breakout, there was a brief period of reverberating shock waves between the samarium sample and lithium fluoride window. After reverberation was complete, pressure remained quasi-static apart from a modest wave reflection at the Al-Sm interface. Eventually shock breakout occurred at the window free surface, causing the apparent velocity to decrease.

**Figure 7:**
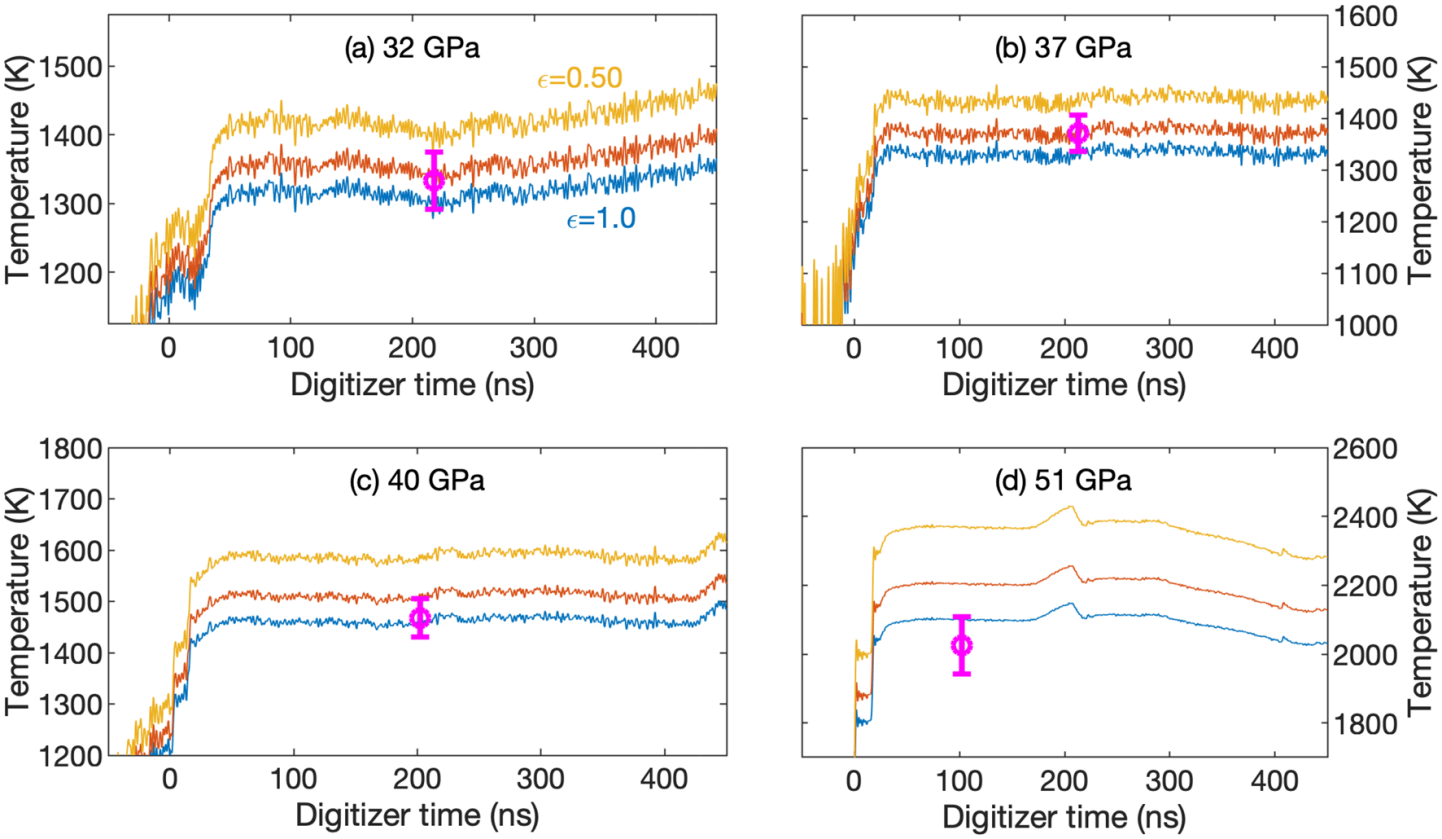
Temperatures calculated from 1125 nm pyrometry signals at emissivities of 1.0, 0.75, and 0.50. Circle symbol and error bars indicate values from Table 2. This is an averaged value over four wavelength bands. The temperature in (d) is lower because the longer wavelength channels give temperatures below the 1125 nm value.

Figure 7 shows temperatures calculated from the 1125 nm pyrometry channel for a range of sample emissivity. At ambient conditions, the near-infrared emissivity of Sm is 0.40–0.55 in a vacuum^18,19^. However, the inevitable formation of a high-index oxide layer increases emissivity to 0.6–0.7 for 1000–1500 nm wavelengths, as discussed in Ref.^20^. As shown in Ref.^21^, the 1125 nm measurements seem to be unaffected by the glue emission. The fact that the averaged temperature values (magenta circles) in Fig 7c, d comes out much lower implies that glue emission is contributing to the overall radiance. Additional pyrometry analysis is reported in the Methods section.

## Discussion

**Figure 8:**
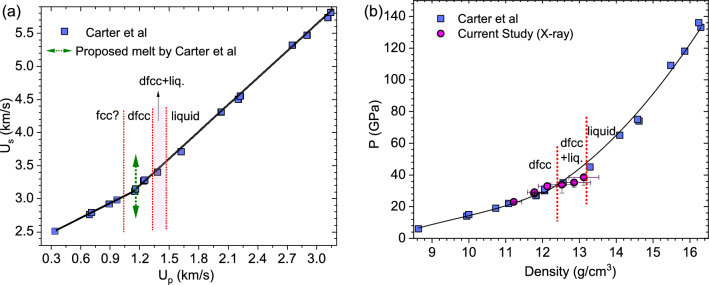
(**a**) Hugoniot in the $U_s-u_p$ and (**b**) pressure-density plane. Square symbols are the data from Carter *et al.*^15^, magenta-filled circles are the densities calculated using x-ray diffraction data. Black solid lines are guide to the eye. The green-dashed arrowed line corresponds to where Carter *et al.*^15^ proposed melting would occur. The red boundaries line are drawn based on the phase boundaries observed in x-ray data from this study.

Figure 8a shows the Hugoniot in the $U_s-u_p$ plane. The green arrowed line ($u_p=1.15$ km/s) corresponds to where Carter *et al.*^15^ have previously interpreted melting to occur based on the kink they observed in the $U_s-u_p$ plot. From our x-ray diffraction data, we have clearly shown that the solid dfcc phase is still stable at 1.08 $\hbox {km/s}< u_p < 1.33$ km/s. Our data suggests that what was reported in previous study to be melting is most likely a solid-solid phase transition. Additionally, for 1.33 $\hbox {km/s}< u_p < 1.47$ km/s, there is a co-existence of solid and liquid phases, followed by a complete melt above 1.47 km/s. More x-ray data are needed to conclude the exact phase boundary of the fcc to dfcc phase transition. Limitations on available sample and ablator thickness at the time of these experiments precluded use of a larger laser spot to drive a steady shock through the sample at the maximum pulse duration. Future experiments can use deposited ablators and samples to probe phase transitions at lower pressures.

The observed d-spacings from the x-ray diffraction data allow for determination of the lattice parameters of the dfcc phase. The resulting unit cell volume in turn enables determination of density. Figure 8b shows the densities calculated from the x-ray diffraction data as a function of pressure (determined through impedance matching using the particle velocity measured at the Sm/LiF interface) together with the densities determined from the impedance matching method. Our data agrees well with the previous study by Carter *et al.*^15^. We observe a small deviation in the density measurements of the dfcc+liquid phase using x-ray diffraction data, likely due to the additional peak broadening resulting from the presence of the liquid phase. The extraction of density from the liquid phase remains a challenge to date.

We note that there is no abrupt change in the density (determined from impedance matching) observed upon liquid transition. This could be due to the fact that the volume change associated with a phase transformation of a material under shock loading conditions is not always obvious through Hugoniot measurements. This can be seen from many previous works on shock compressed metals, such as, copper^22^, cerium^23^, and aluminum^24^. In the case of copper, Hayes *et al.*^22^ do not observe discontinuity along the Hugoniot. However, sound speed measurements on the other hand show a clear discontinuity upon melting. Therefore, x-ray diffraction or sound speed measurements are critical to pin down the exact phase transition stress, underscoring the significance of the current study in determining the phase boundary.

Our study shows that the dfcc phase is stable at higher temperatures between the pressure of 23-33 GPa, followed by a mixture of dfcc+liquid (33 to 38 GPa). Melting begins at 1333 K and is complete above 1468 K. The onset of melting we observe in our current study is 800 K lower temperature than that of Errandonea *et al.*^14^ DAC heating study. In their study, melting was identified visually by the motion in the interference pattern created on the sample surface by Ar laser radiation using a 30 µm diameter hotspot created by defocused laser beams^14,25^. Needless to say that there could be quite a bit temperature gradient from the unheated sample. In fact, a careful study done by Karandikar *et al.*^26^ on Ta has shown that the previous melting reported by Errandonea *et al.*^27^ using similar methods as they employed on Sm has shown a 500 K discrepancy on Errandonea’s melting curve. It is quite likely that the temperature measurements were taken upon completion of the melt, hence, the upper end of the melting curve is measured. The much lower melting we observed in our study posits that more careful high temperature studies are needed in order to further constrain the melting curve of samarium.

**Figure 9:**
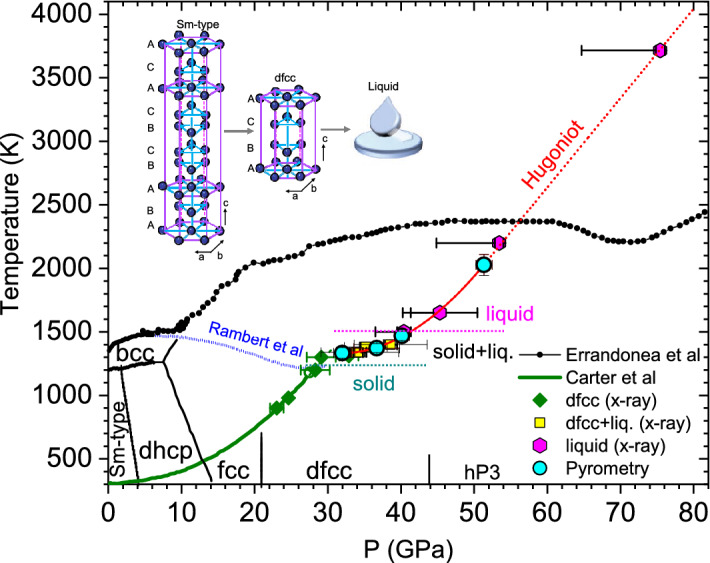
Proposed phase diagram of Samarium showing the solid-liquid phase boundaries. The uncertainties in pressures are presented in Table 1. Cyan circles are measured temperatures from pyrometry measurements. Green diamonds represent the solid dfcc phase, the yellow squares are the dfcc+liquid phase, and the magenta hexagons are the liquid phase from current XRD study. Small filled circles indicate the DAC melt curve proposed by Errandonea *et al.*^14^. The solid black lines are phase boundaries from various DAC studies^11,13^. Blue dashed line is the proposed melting curve by Rambert et al^13^. The green curve is the Hugoniot from a previous shock study^15^.

Figure 9 plots the measured temperature data together with pressures obtained from the velocimetry measurements in the existing phase diagram of samarium. We have also overlaid the combined pressure and structural information obtained from our x-ray diffraction data. Note that only pressure was measured in those experiments, temperature information were obtained from Carter *et al.*study^15^ and this current study. Our results show a plateau between 30–38 GPa, consistent with the coexistence of dfcc+liquid phase. Melting begins at 1333 K and is complete above 1468 K. Upon completion of melt, there is a monotonous increase in temperature with increase in pressure. Our observed melting in current study is 800 K lower than that reported by the Errandonea *et al.*DAC study. Based on what we observe in our current study, it is likely that the melt curve of samarium has a negative slope as previously proposed by Rambert *et al.*^13^ (blue dashed line). Interestingly, similar trend of decreasing melt temperature with increase in pressure has been observed in other metals, such as, cerium^23^ and lithium^28^.

## Conclusions

Our study provides benchmark data to tightly constrain first-principles simulations for very complex *f*-electron systems. Coupling shock compression with x-ray diffraction, we demonstrated the existence of the solid Sm dfcc phase above 23 GPa, which remains stable up to 33 GPa along the Hugoniot. Our XRD data combined with the temperature measurements establishes the onset of melting in Sm at 33 GPa, with liquid-solid coexistence up to 38 GPa. High-quality x-ray diffraction data indicate complete shock melting by 40 GPa. Our study provides key insights into understanding the phase behavior of Sm along the Hugoniot. Experimental data—more specifically, in-situ x-ray diffraction data and temperature measurements—are paramount to constrain these first-principles calculations and develop material models.

**Table 1:** Experimentally determined lattice parameters and Hugoniot states of shocked Sm. The volume is calculated using the lattice parameters obtained from x-ray diffraction measurements. Therefore, the volume for the liquid phase is not determined as extraction of volume for liquid data still remains a challenge. Density ($\rho$), and pressure (*P*) are determined from the Hugoniot jump conditions and *Monte Carlo* impedance matching technique. The initial density was 7.536 g/cm$^3$ with an uncertainty of 0.3%. The upper bound of the pressure error is from impedance matching and lower bound is from incremental impedance matching. On no shots did the shock pressure, at times later than the initial breakout into the LiF window, exceed the initial pressure from impedance matching.

Shot ID	c/a ratio	Volume/atom (Å$^3$)	Sm/LiF $u_p$ (km/s)	$u_p$ (km/s)	$U_S$ (km/s)	$\rho$ (g/cm$^3$)	*P* (GPa)
s55	2.459 ± 0.024	19.93 ± 0.48	1.18±0.03	1.08 ± 0.03	3.06 ± 0.03	11.68 ± 0.08	23.02 (+0.86,-0.91)
s18	2.482 ± 0.013	21.30 ± 0.22	1.31±0.02	1.19 ± 0.02	3.20 ± 0.02	12.04 ± 0.05	28.29 (+0.62,-1.99)
s57	2.486 ± 0.022	21.19 ± 0.35	1.33±0.04	1.20 ± 0.03	3.23 ± 0.04	12.09 ± 0.10	29.14 (+1.19,-2.01)
s17	2.491 ± 0.026	20.58 ± 0.41	1.41±0.03	1.27 ± 0.03	3.36 ± 0.01	12.30 ± 0.08	32.88 (+0.29,-0.91)
s220	2.497 ± 0.032	19.29 ± 0.48	1.48±0.06	1.35 ± 0.01	3.40 ± 0.06	12.52 ± 0.15	33.87 (+1.92,-5.29)
s90	2.517 ± 0.045	19.41 ± 0.66	1.52±0.04	1.36 ± 0.02	3.44 ± 0.04	12.57 ± 0.08	35.18 (+1.17,-2.93)
s181	2.523 ± 0.042	19.02 ± 0.59	1.62±0.02	1.45 ± 0.01	3.55 ± 0.06	12.60 ± 0.47	38.56 (+0.90,-4.99)
s146	—	—	1.68±0.03	1.50 ± 0.02	3.62 ± 0.11	12.73 ± 0.43	40.39 (+0.95,-3.90)
s129	—	—	1.73±0.03	1.61 ± 0.02	3.77 ± 0.06	13.02 ± 0.33	45.32 (+4.02,-5.10)
s235	—	—	2.03±0.01	1.79 ± 0.01	4.00 ± 0.05	13.49 ± 0.19	53.40 (+0.58,-8.58)
s329	—	—	2.58±0.02	2.22 ± 0.01	4.56 ± 0.05	14.50 ± 0.20	75.43 (+0.62,-10.75)

**Table 2:** Experimentally determined pressure and temperature states of shocked Sm. Values are averaged during the quasi-static period in each case except Shot 5, which was restricted to $t < 160$ ns. Parenthesis indicate temperatures obtained from the 1125 nm band only; all other temperatures are based on three spectral bands.

Experiment	Interface velocity (km/s)	Temperature (K)	Pressure (GPa)
Shot 1	1.43 ± 0.01	1330 ± 40	31.9 ± 1.2
Shot 3	1.56 ± 0.01	1370 ± 40	36.7 ± 3.1
Shot 4	1.63 ± 0.02	1470 ± 40	40.1 ± 1.0
		$$\left( 1510^{+60}_{-50} \right) $$	
Shot 5	1.98 ± 0.03	2030±80	51.3 ± 1.1
		$$\left( 2200^{+200}_{-100} \right) $$	

## Methods

### Diffraction experiments

Diffraction experiments were performed at the Dynamic Compression Sector (DCS) at the Advanced Photon Source, using the 100 J laser^3,4^ as a driver. We have performed *in situ* x-ray diffraction measurements in Sm using  23.54 keV x-rays. The two-dimensional x-ray diffraction rings were integrated azimuthally using DIOPTAS^29^ and FIT2D^30^ with poly-crystalline silicon as a calibrant to obtain one-dimensional diffraction profiles. The resulting one-dimensional profiles were then analyzed using Rietveld methods or by fitting the d spacings of individual diffraction peaks using a combination of various software, including GSAS-II^31^, VESTA^32^ and CRYSTALMAKER^33^.

Our targets were planar stacks consisting of a 50 $\upmu \hbox {m}$ aluminized polyimide (Dupont Kapton HN) ablator, nominally 15- or 25 $\upmu \hbox {m}$ Sm samples (99% purity, purchased from Goodfellow Corporation, $\rho _0 = 7.536$ $\hbox {g/cm}^3$) and a LiF window (8 mm x 4mm x 1.5 mm thick) purchase from Asphera). We measured the thicknesses of the Sm samples to 1 $\upmu \hbox {m}$ accuracy with a Nikon MF-501 drop micrometer. The LiF windows were coated with a 100 nm thick Al mirror (2 mm x 2mm square) on the sample side and 532 nm AR coating on the vacuum side. We assembled the targets using AngstromBond 9110LV and placed in a 10-ton press until the epoxy had fully cured.

### Temperature experiments

Near-infrared optical pyrometry was used to infer temperature. Symmetric impact of a 25 mm diameter, 2 mm thick aluminum flyer with an identical buffer created an initial shock wave. A 15 mm diameter, 0.5 mm thick samarium sample was glued the buffer with AngstromBond, followed by a 25 mm diameter, 2 mm thick LiF window attached with Loctite 326 glue. Aluminum coating (front side) and black paint (outer edges and back side) minimizes stray light reaching the pyrometry probe, where a bundle of angle-polished optical fibers were imaged onto the sample. Four pyrometry measurements (825/1125/1225/1425 nm center, 50 nm full-width half-maximum) were preformed with low-OH multi-mode fibers using avalanche indium gallium arsenide photodiodes.

**Figure 10:**
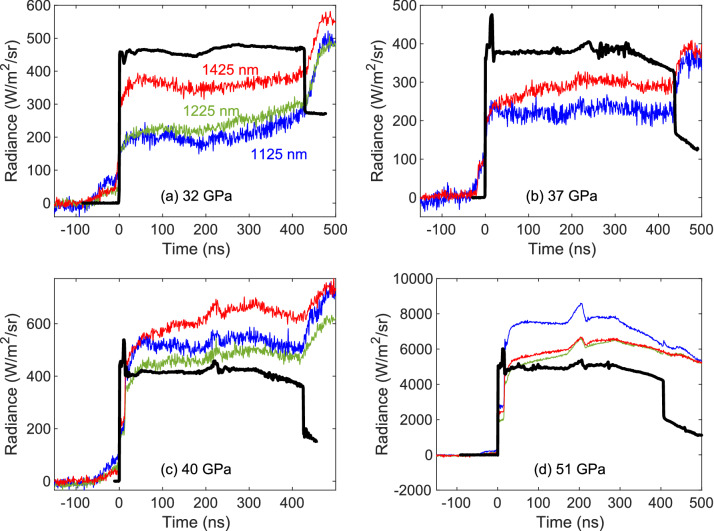
Measured apparent PDV velocities (in black) and pyrometry data centered at 1425 nm (in red) for four shots. Green curves indicate a 1225 nm measurement available in all but one experiment. Black lines show a scaled version of the velocimetry measurement for comparison. Time zero corresponds to shock breakout time at Sm/LiF interface.

Pyrometry signals were converted to radiance via black body (Mikron Infrared, Inc., model M390) calibration immediately before each experiment. Wavelength bands below 1000 nm were omitted from further analysis due to an obvious light flash in every experiment at the shock breakout. Figure 10 shows the band radiance obtained from the InGaAs signals after calibration. The results are similar to velocity at low pressure, but there is a noticeable upward trend in the 1225/1425 nm bands, particularly above 40 GPa. We suspect this rise is caused by decomposition of glue between the Sm sample and LiF window, which does not seem to affect the 1125 nm measurement, similar to reports in Ref.^21^. Measured radiance also increased at breakout, presumably due to fracture luminescence^34^.

The Metropolis-Hastings approach described in Ref.^20^ was used to further analyze steady-state temperature. Constant emissivity was assumed in each spectral band ($>1000$ nm only), with values ranging from 0.1 to 1. The results indicate emissivities largely in the range of 0.4–0.7, decreasing as a function of wavelength, consistent with ambient Sm measurements^18,19^. The combined results are generally consistent with Fig. 7 except at the highest pressures, where the longer wavelength channels pull temperature below the 1125 nm value. Table 2 reports two temperatures for the higher pressure experiment: one obtained from Metropolis-Hastings analysis of three spectral bands and the other based a single band with $epsilon=0.50-1.00$ emissivity.

### Pressure-determination

Time-resolved optical interferometry was used all experiments: Velocity Interferometer System for Any Reflector (VISAR)^35^ with diffraction and Photonic Doppler Velocimetry (PDV)^36^ with temperature. The former operates at 532 nm, while the latter operates at 1550 nm. Both measurements track motion of the Sm-LiF interface.

**Figure 11:**
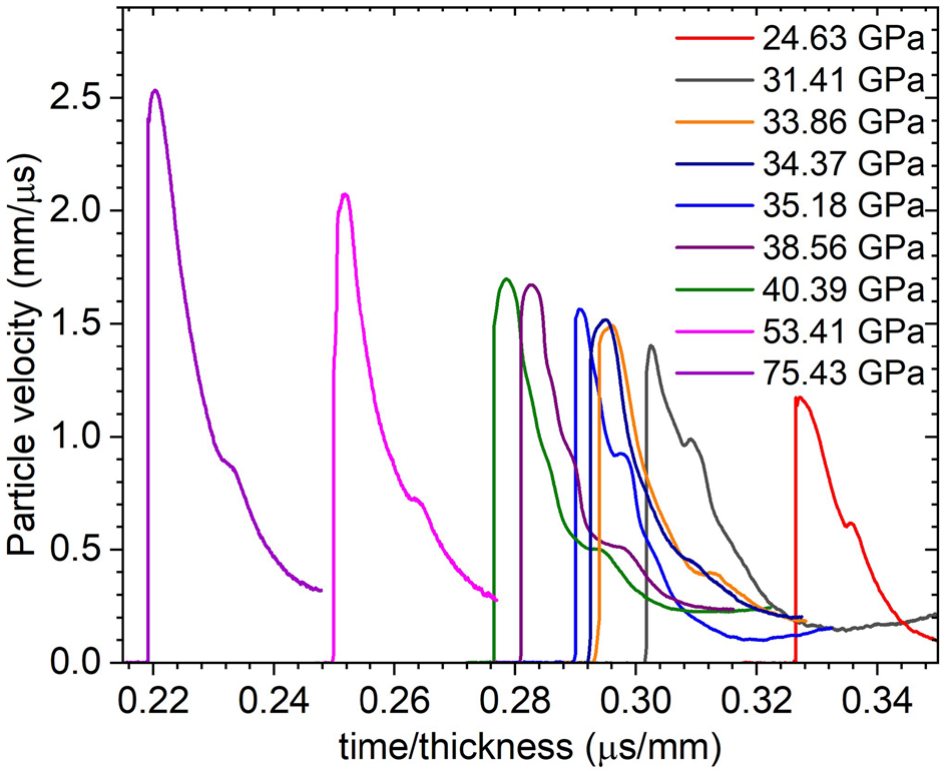
x-t diagram for selected shots showing the pressure variation across the sample for 15 and 25 $\upmu \hbox {m}$ thick samples.

VISAR measurements used Velocity per Fringes (VPFs) of 1.555 and 0.945 km/s. Ablation of the Kapton layer generated a shock that propagated into the Sm sample. Particle velocity measured at the Sm/LiF interface is linked to pressure by impedance matching to the previously reported Hugoniot for LiF^37^ and Sm^15^. The peak of the particle velocity traces (Fig. 11) were used for impedance matching, combined with a Monte-Carlo method to determine pressure uncertainties. Results are shown in Table 1.

PDV measurements used a frequency-shifted configuration to maintain several fringes over the 5 ns analysis duration. Three SMF-28 fibers in the imaged bundle provided redundant data to compensate for dynamic speckle effects. Quasi-steady values were used for impedance matching.

### Pressure-distribution analysis

**Figure 12:**
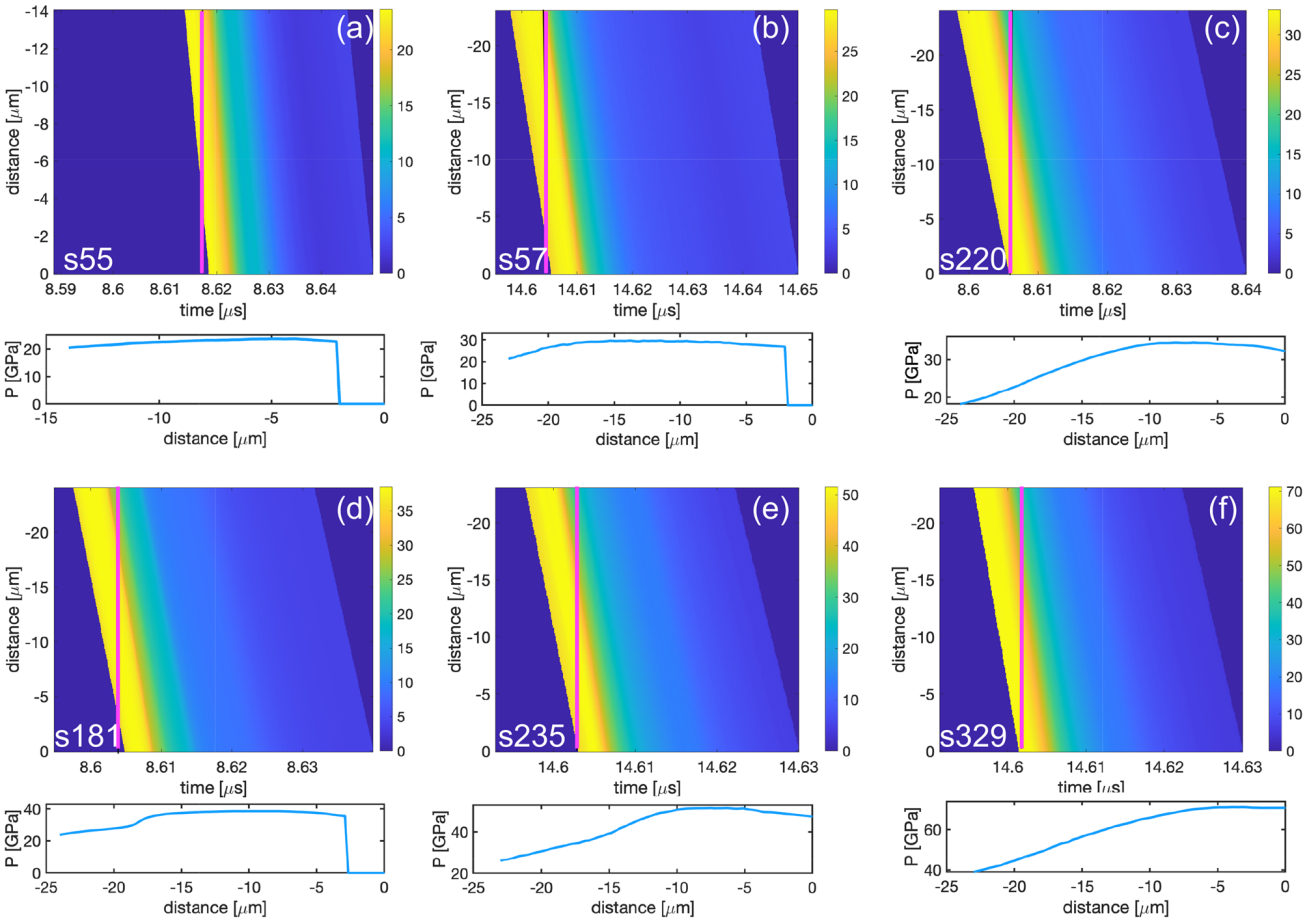
x-t diagram for selected shots showing the pressure variation across the sample for 15 and 25 $\upmu \hbox {m}$ thick samples. Magenta line represents the time x-ray diffraction was collected. Bottom panel shows the pressure gradient across the sample position at the time of x-ray collection.

To understand the pressure distribution in the sample at the time of the x-ray image, we have used the incremental impedance matching method to back-propagate the stress within the sample^38,39^. In this technique, the wave profile measured at the Sm/LiF interface is used to determine an in-situ velocity in the Sm sample. This velocity can be related to waves propagating through the Sm sample by the method of characteristics, similar to that done in the Inverse Lagrangian Analysis^40^. By propagating the waves backwards using a compressed sound velocity, we were able to construct a map of pressure as a function of time and distance in the samples. From the pressure map, we could extract the pressure in the sample at the time of the x-ray image. Pressure maps and corresponding sample pressure distributions at the time of x-ray exposure are shown in Fig. 12. We used this method to obtain the lower error bound of the pressure in Table 1.

From the sample pressure distributions, an overtaking wave from the front surface has propagated into the sample and released pressure by the time that the x-ray image was taken. This is especially noticeable for the highest-pressure shots (s235 and s329), where the 1 $\sigma$ standard deviation in pressure exceeds 8 GPa. As both shots reached pressures to fully melt the Sm sample, the decay in pressure should not impact the phase identified from the x-ray analysis because the entire sample was shocked to the peak state determined from impedance matching prior to decaying. In the $P-T$ plane shown in Fig. 9, the release curve from these peak states do not cross the melt curve, so solidification would not be expected to occur.

## Data Availability

The datasets generated during and/or analysed during the current study are available from the corresponding author on reasonable request.
